# Hygroscopic compounds in spider aggregate glue remove interfacial water to maintain adhesion in humid conditions

**DOI:** 10.1038/s41467-018-04263-z

**Published:** 2018-05-22

**Authors:** Saranshu Singla, Gaurav Amarpuri, Nishad Dhopatkar, Todd A. Blackledge, Ali Dhinojwala

**Affiliations:** 1 Department of Polymer Science, The University of Akron, Akron, OH 44325 United States; 2 Department of Biology, Integrated Bioscience Program, The University of Akron, Akron, OH 44325 United States; 3 Present Address: Eastman Chemical Company, Corporate Analytical Division, Kingsport, TN 37662 United States; 4 Present Address: Avery Dennison Polymers, Adhesives and Coatings Center of Excellence, Mill Hall, PA 17751 United States

## Abstract

Adhesion in humid environments is fundamentally challenging because of the presence of interfacial bound water. Spiders often hunt in wet habitats and overcome this challenge using sticky aggregate glue droplets whose adhesion is resistant to interfacial failure under humid conditions. The mechanism by which spider aggregate glue avoids interfacial failure in humid environments is still unknown. Here, we investigate the mechanism of aggregate glue adhesion by using interface-sensitive spectroscopy in conjunction with infrared spectroscopy. We demonstrate that glycoproteins act as primary binding agents at the interface. As humidity increases, we observe reversible changes in the interfacial secondary structure of glycoproteins. Surprisingly, we do not observe liquid-like water at the interface, even though liquid-like water increases inside the bulk with increasing humidity. We hypothesize that the hygroscopic compounds in aggregate glue sequester interfacial water. Using hygroscopic compounds to sequester interfacial water provides a novel design principle for developing water-resistant synthetic adhesives.

## Abstract (web summary)

Spider aggregate glue avoids failure in humid environments but the fundamental mechanism behind it is still unknown. Here, the authors demonstrate that humidity-dependent structural changes of glycoproteins and sequestering of liquid water by low molecular mass compounds prevents adhesion failure of the glue in humid environments.

## Introduction

Most synthetic adhesives fail interfacially at humidities above a critical value when water lubricates the contact interface, resulting in weakening of adhesive–substrate interactions and build up of interfacial swelling stresses^1–4^. Nature, on the other hand, contains a multitude of biological adhesives used by various organisms in high humidity or underwater environments for prey capture, self-defense, and nest building^5, 6^. Aggregate glue is used by orb web spiders in their sticky capture spirals to catch and retain prey^7–11^. Different species of spiders forage in diverse habitats, ranging from dry to humid, and aggregate glue adhesion is humidity-responsive such that adhesion is maximized for a given species at humidity matching its preferred microhabitat^12^. Some species that forage in wet habitats like *Tetragnatha*^12^ and *Cyrtarachne*^13^ produce aggregate glue that consistently adheres better at humidities exceeding 90% RH, contrary to most synthetic adhesives^1, 2^. Even the aggregate glue from species found in dry habitats shows cohesive failure, instead of interfacial failure, at high humidity^12^. Thus, spider aggregate glue is a model adhesive system for avoiding interfacial failure under humid conditions; chemistry and mechanism can be applied to the adhesive design.

Aggregate glue self-organizes into regularly spaced droplets (beads) on the underlying flagelliform thread (string) due to Rayleigh instability, commonly referred to as a beads-on-a-string (BOAS) morphology^9, 14^. The aggregate glue is comprised of two glycoproteins—aggregate spider glue 1 and 2 (ASG1 and ASG2) along with a cocktail of low molecular mass organic and inorganic compounds (collectively referred to as LMMCs) and water^9, 10, 14–19^. The glycoproteins and LMMCs act synergistically to generate humidity-responsive adhesion behavior of aggregate glue^20^ with adhesion maximized at humidity close to each spider species’ foraging habitat humidity^12^. ASG1 includes amino acid sequences similar to chitin-binding domains with high proportions of charged amino acids, while ASG2 shows similarities to elastin and flagelliform spider silk repetitive domains that are critical for extensibility and elasticity of the glue droplet^16^. Both ASG1 and ASG2 are O-glycosylated primarily by N-acetylgalactosamine, and small amounts of other sugars such as galactose, mannose, and fucose^9, 10, 18, 21–23^. While many studies attribute the adhesion of glue droplets to glycoproteins, no direct proof is evident from the literature^7, 14, 24–27^. The LMMCs, on the other hand, include small polar aliphatic compounds such as GABamide, N-Acetylputrescine, Isethionic acid, and others; which play a dual role of solvating the glycoproteins and sequestering atmospheric water that plasticizes the aggregate glue, making it soft and tacky^9, 14, 15, 17, 20^. Understanding how glycoproteins and LMMCs interact at the interface is crucial for providing insights into the fundamental mechanism of spider aggregate glue adhesion at high humidity.

Here, we perform humidity-dependent sum frequency generation spectroscopy (SFG) measurements for *Larinioides cornutus* spider aggregate glue in contact with sapphire substrate. SFG is a non-invasive and inherently interface-specific technique, providing information about the molecular groups of spider aggregate glue in close vicinity of the substrate^28–32^. The in situ humidity setup in conjunction with SFG allows the monitoring of changes occurring at the spider aggregate glue/sapphire interface as a function of humidity. *Larinioides cornutus* was chosen as the subject of study because its aggregate glue maximizes adhesion at intermediate humidity and undergoes cohesive failure at high humidity^12, 33^. We provide direct evidence for exclusion of liquid-like water at the contact interface by LMMCs at high humidity, so that the spider aggregate glue maintains its interfacial adhesion even at extreme humidity. We demonstrate that the glycoproteins present at the contact interface rearrange at the interface with humidity and interact with LMMCs to enable spider aggregate glue’s smart adhesive properties.

## Results

### Immobilizing the capture silk glue

**Fig. 1:**
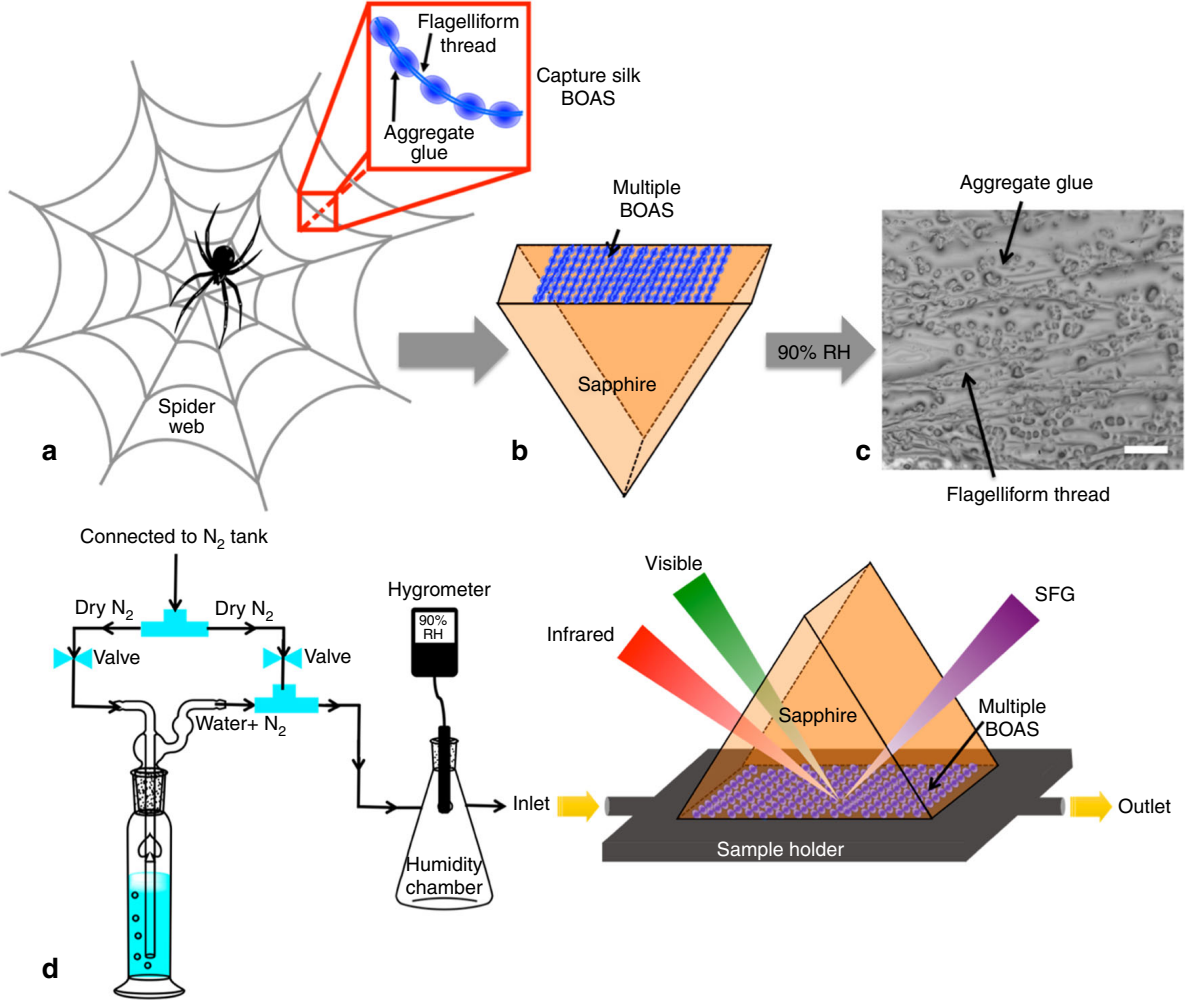
Immobilizing the capture silk glue. Multiple capture silk beads-on-a string (BOAS) strands, comprised of aggregate glue droplets (beads) supported on an underlying flagelliform thread (string), are collected from 2–3 webs of *Larinioides cornutus* and deposited on a clean sapphire prism (**a**, **b**). The sapphire prism with multiple BOAS strands is exposed to high humidity to allow complete spreading of aggregate glue droplets, as depicted in the optical microscope image (**c**). Scale bar is 100 μm. The sapphire prism with multiple BOAS strands is mounted onto a sample holder and the whole assembly is connected to a humidity setup for collecting SFG spectra at different relative humidities, the schematic of which is shown in panel **d**

Orb web spiders such as *Larinioides cornutus* use the sticky capture silk to catch and retain prey in their webs. The capture silk is an assembly of micron-sized aggregate glue droplets (beads) regularly spaced on an underlying flagelliform thread (string), commonly referred to as BOAS morphology (Fig. 1a)^9, 14^. We collected multiple BOAS strands from 2–3 spider webs and adhered them to the sapphire prism visually in a parallel configuration to cover as much of the sapphire surface as possible (Fig. 1b). The sapphire prism with multiple BOAS strands was then exposed to high humidity using either D_2_O or H_2_O vapors to allow spreading of aggregate glue droplets (facilitated by reduction in viscosity at high humidity), resulting in the formation of a uniform spider aggregate glue layer (shown in Fig. 1c)^12^. The sapphire prism with multiple BOAS strands was then mounted onto a sample holder and the whole assembly was connected to a humidity setup (Fig. 1d) for probing the contact interface between BOAS and sapphire substrate as a function of humidity. Both D_2_O and H_2_O were used here to help in identifying changes in the interfacial structure as a function of humidity. Although the BOAS consist of both aggregate glue droplets and the flagelliform thread, we do not expect the flagelliform thread to contribute to the obtained SFG spectra because the flagelliform thread is covered with aggregate glue throughout its length, and it is the the aggregate glue that primarily makes contact with the sapphire substrate^12, 33^. Also, the thickness of the aggregate glue layer is of the order of a few microns, whereas the probe depth of SFG is only a few nm^7, 12^. Thus, the aggregate glue layer primarily contributes to the obtained SFG spectra. We collect SFG spectra for the aggregate glue in its native state (referred to as pristine aggregate glue) at low (10% RH) and high (90% RH) humidity in PPP, SSP, and PSP polarizations, where the three indices represent the polarizations of SFG, visible, and IR beams, respectively. The different polarizations can be used to infer the molecular orientation. We use the SFG results at low humidity (10% RH) to understand the different peak assignments, then follow how spectral features change with humidity.

### Glycoproteins present at the contact interface

**Fig. 2:**
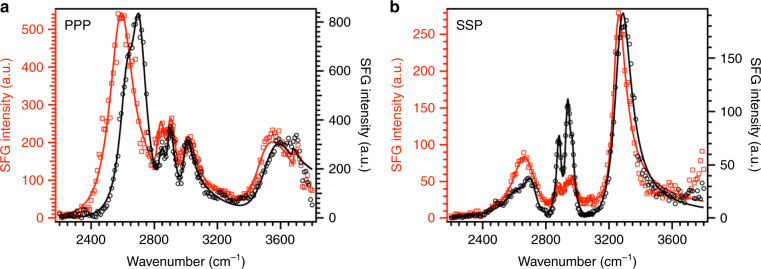
SFG spectra for pristine and washed aggregate glue. SFG spectra for pristine aggregate glue/sapphire (red empty squares) and washed aggregate glue/sapphire (black empty circles) interfaces collected at low humidity (10% RH) in PPP (**a**) and SSP (**b**) polarizations. The solid lines are a fit to the Lorentzian equation. Pristine aggregate glue consists of both glycoproteins and LMMCs, while washed aggregate glue consists of predominantly glycoproteins (Supplementary Fig. 2)^20, 45^. The similarity between the pristine and washed aggregate glue SFG spectra suggests the presence of glycoproteins at the aggregate glue/sapphire contact interface. The slight differences in the hydrocarbon peaks are not discussed due to difficulty in de-convoluting the hydrocarbon peaks

The SFG spectrum for pristine aggregate glue/sapphire interface collected with D_2_O vapors in PPP polarization at low humidity is shown in Fig. 2a (component peaks shown in Supplementary Fig. 1 and Supplementary Note 1). The spectrum consists of peaks at ~2580 and ~2700 cm^−1^ which are assigned to the O–D stretching modes of D_2_O molecules with one hydrogen bonded O–D and one non-hydrogen bonded (or dangling) O–D, respectively^3, 34–36^. The two peaks indicate the presence of low coordination water (or less hydrogen bonded water) at the contact interface. We do not observe liquid-like water peaks in the 2400–2500 cm^−1^ region at 10% RH. The use of D_2_O helps in minimizing the interference between hydrocarbon and water vibrational modes. However, it brings in the complexity of H/D exchange for O–H groups of sugar residues present in the glycoproteins and O–H groups of sapphire surface, which may also contribute to the peaks observed in D_2_O region^37–41^. The exchange of sapphire O–H to O–D is controversial in the literature^39, 40^, but previous work done in our group suggests that the sapphire O–H does not readily exchange to O–D^41^. Our results are consistent with the previous studies^41^ because we observe a broad peak around 3550 cm^−1^ that is attributed to the O–H vibration of sapphire hydroxyl groups in contact with pristine aggregate glue. When sapphire O–H groups are in contact with air (air/sapphire interface), a sharp peak at ~3720 cm^−1^ is observed for the sapphire hydroxyl groups. However, when sapphire O–H groups come in contact with other molecules such as poly(methyl methacrylate) or polystyrene, the O–H peak becomes broader and shifts to lower wavenumbers due to acid–base interactions between the sapphire O–H groups and the other molecules in contact^29, 42^. The amount of red-shift is related to the interaction strength according to the Badger–Bauer equation; the higher the shift, the stronger the interaction strength. The calculated interaction strength between pristine aggregate glue and sapphire O-H groups (Supplementary Table 1) is comparable to the strongest interaction between carbonyl groups and sapphire surface O–H groups reported in literature^29, 42^. We also observe C–H stretching vibrations in the 2800–3050 cm^−1^ region. The hydrocarbon peaks are difficult to de-convolute due to the possibility of multiple assignments, thus we do not discuss the hydrocarbon peaks. The corresponding spectrum for pristine aggregate glue/sapphire interface collected at 10% RH achieved using H_2_O vapors is shown in Supplementary Fig. 3, which consists of peaks around 3560 and 3660 cm^−1^. The two peaks could arise either due to the presence of loosely coordinated water or due to sapphire O–H groups in contact with pristine aggregate glue (Supplementary Note 3).

The SFG spectrum for pristine aggregate glue/sapphire interface at low humidity in SSP polarization (Fig. 2b) consists of peaks at ~2650 and ~2730 cm^−1^ in the O–D stretching vibration region (similar to those in PPP), signifying the presence of low coordination water (or less hydrogen bonded water)^3, 34–36^. In addition to the O–D peaks, we observe a very strong peak at ~3260 cm^−1^ which is assigned to the N–H stretch, as reported in literature^30–32, 43, 44^. Two possible assignments for the N–H peak are: (1) glycoproteins and (2) certain LMMCs (Isethionic acid and GABAmide)^18^. To understand the origin of the N–H peak, the sapphire prism with pristine aggregate glue is washed with Millipore water to remove the water soluble LMMCs and leave washed aggregate glue containing only the glycoproteins (Supplementary Note 2)^20, 45^. We validate the efficacy of washing by using Raman spectroscopy. As expected, the washed aggregate glue Raman spectrum shows only protein-specific peaks and not LMMC-specific peaks (Supplementary Fig. 2)^45^. We collect SFG spectra for both washed aggregate glue and washed extract containing only LMMCs (referred to as LMMC-extract) to determine the origin of N–H peak; the results are discussed in the following paragraphs.

The washed aggregate glue SFG spectrum collected in PPP and SSP polarizations is shown in Fig. 2a, b, respectively. The washed aggregate glue/sapphire SFG spectrum in PPP polarization at 10% RH (Fig. 2a) consists of low coordination D_2_O peaks at ~2650 and ~2700 cm^−1^, which are blue-shifted relative to those observed for pristine aggregate glue^34–36, 46^. The blue-shift in D_2_O peaks could be related to absence of hygroscopic LMMCs in washed aggregate glue. Along with D_2_O peaks, we observe the shifted sapphire O–H peak at ~3550 cm^−1^^29, 42^. The SFG spectra for the pristine and washed aggregate glue are similar except for subtle differences in the 2200–2800 cm^−1^. The similarity between pristine and washed aggregate glue SFG spectra prevails in the SSP polarization (Fig. 2b), where a strong N–H peak at ~3270 cm^−1^ is observed along with low coordination D_2_O peaks^30–32, 43, 44^. The corresponding spectra collected for washed aggregate glue/sapphire interface using H_2_O vapors are presented in Supplementary Fig. 4. The similarity between SFG spectra of pristine and washed aggregate glue (devoid of LMMCs) indicates the presence of glycoproteins at the contact interface. Further SFG spectra collected for drop-cast LMMC-extract film (Supplementary Fig. 5 and Supplementary Note 4) concur with our findings as the N–H peak is absent in SSP spectrum for LMMC-extract/sapphire interface.

**Fig. 3:**
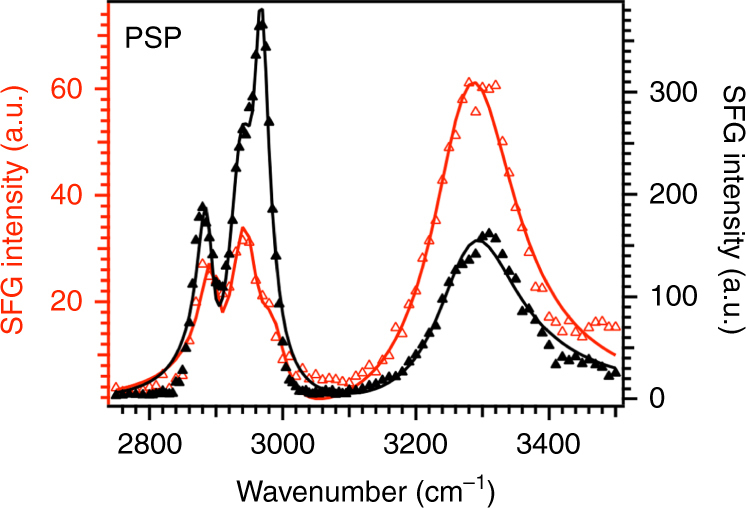
Chiral SFG spectra for pristine and washed aggregate glue. SFG spectra collected in PSP polarization for pristine aggregate glue/sapphire (red empty triangles) and washed aggregate glue/sapphire (black filled triangles) at 10% RH. Solid lines are a fit to the Lorentzian equation

-To confirm the presence of glycoproteins at the contact interface, we collect SFG spectra for both pristine and washed aggregate glue in PSP polarization at 10% RH (Fig. 3), since this polarization is selective to the presence of chiral protein secondary structures such as α-helices or β-sheets^30, 44, 47^. The SFG spectra for both pristine and washed aggregate glue in PSP show a N–H peak at ~3270 cm^−1^. According to previous studies done by Yan et al.^30, 44^, the presence of a N–H stretch peak in PSP polarization is associated with the presence of α-helices or β-sheets at the contact interface. Additional SFG spectra in the amide I region are required to determine the absolute nature of secondary structures; collection of these spectra  is not possible due to attenuation of infrared beam by the sapphire substrate above 5 μm wavelength (or below 2000 cm^−1^ wavenumber)^48^. Our observations of protein secondary structures at the interface establish that glycoproteins are primarily involved in the adhesion of spider aggregate glue directly to the surface, as both ASG1 and ASG2 contain α-helix and β-sheet forming domains^16, 19^. Future studies are required to determine the absolute nature of glycoprotein secondary structures at the interface by collecting SFG spectra in the amide I region.

### Effect of humidity at the contact interface

**Fig. 4:**
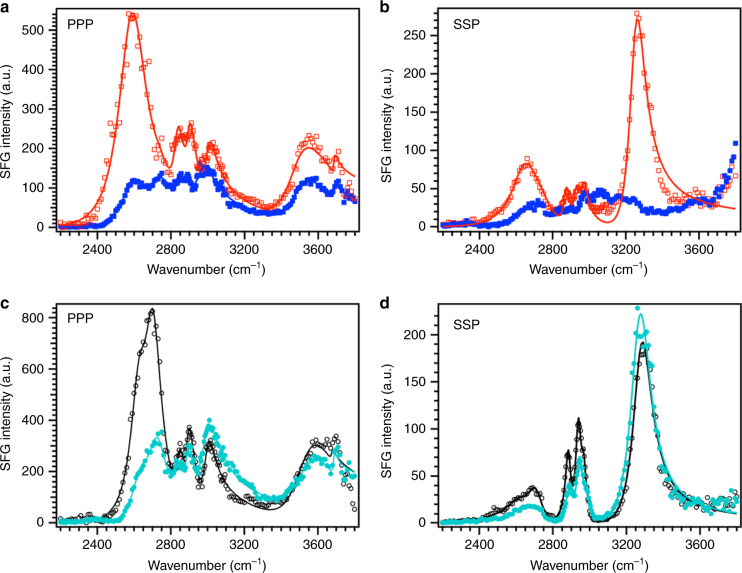
Effect of humidity at the contact interface. SFG spectra for pristine aggregate glue/sapphire at low (red empty squares) and high (blue filled squares) humidity collected in PPP (**a**) and SSP (**b**) polarizations. Similar spectra are collected for washed aggregate glue/sapphire in PPP (**c**) and SSP (**d**) polarizations at low (black empty circles) and high (blue filled circles) humidity. The solid lines are a fit to the Lorentzian equation (Supplementary Tables 3 and 4)

**Fig. 5:**
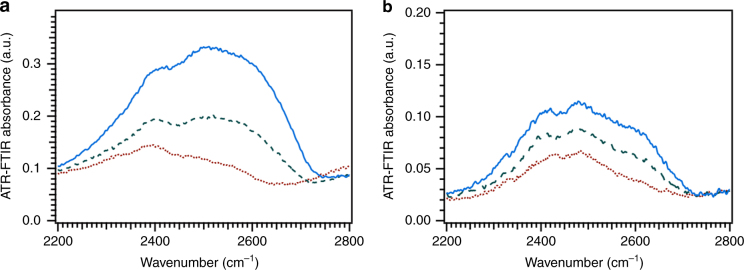
Effect of humidity in the bulk. ATR-IR spectra of *Larinioides cornutus* pristine (**a**) and washed (**b**) aggregate glue at 10% RH (red dotted curve), 50% RH (green dashed curve), and 90% RH (blue solid curve). The pristine aggregate glue spectrum shows a significant change in the D_2_O region (2200–2800 cm^−1^) relative to washed aggregate glue with increasing humidity. All spectral changes occurring with changing humidity are reversible

Figure 4a, b show SFG spectra for pristine aggregate glue/sapphire at low and high humidity in PPP and SSP polarizations, respectively. The SFG spectrum for pristine aggregate glue at low humidity in PPP polarization consists of low coordination water peaks^3, 34–36^ and the broad shifted sapphire O–H peak^29, 42^. At high humidity, the SFG spectrum for pristine aggregate glue consists of peaks at ~2600, ~2730 cm^−1^ and the shifted sapphire O–H peak at ~3550 cm^−1^. Surprisingly, we do not observe any liquid-like D_2_O peaks between 2400–2500 cm^−1^ at high humidity, although previous water uptake measurements show a 100% increase in the volume of the aggregate glue droplets as the relative humidity is increased from 10 to 90%^12, 33^. The liquid-like water peaks are not observed even when H_2_O vapors are used to humidify the pristine aggregate glue (Supplementary Fig. 3). To ensure that the absence of liquid-like water is not an artifact of the humidity setup, we perform similar experiments with polyacrylic acid, a model hygroscopic polymer having ~25% water uptake (Supplementary Note 5). Intriguingly, we observe liquid-like water at the polyacrylic acid/sapphire interface at high humidity (Supplementary Fig. 6) in contrast to pristine aggregate glue. The liquid-like water was also absent for the hydrophobic polyurethane/sapphire interface at high humidity as shown in the previous work^3^. We further collect attenuated total reflectance infrared spectroscopy (ATR-IR) spectra to observe changes in the D_2_O peaks within the bulk of pristine aggregate glue under increasing humidity (Supplementary Note 6). In contrast to the contact interface, liquid-like water (~2400–2500 cm^−1^) increases with increasing humidity in the bulk of pristine aggregate glue (Fig. 5a). However, no significant changes are observed in the bulk of pristine aggregate glue in the amide I and II (1500–2000 cm^−1^) and C–H/N–H stretch (2800–3800 cm^−1^) regions (Supplementary Fig. 7). The broad peak at ~3550 cm^−1^ (Fig. 4a), representative of bonding between the pristine aggregate glue and sapphire substrate, persists at high humidity. The interaction energy calculated using the Badger–Bauer equation changes minimally with increasing humidity (Supplementary Table 1)^29, 42^, thus suggesting that the bonds between the pristine aggregate glue and sapphire are preserved at high humidity.

In SSP polarization, the N–H peak at ~3260 cm^−1^ diminishes at high humidity (Fig. 4b) in comparison to low humidity. The diminished N–H peak indicates changes in the secondary structure of the glycoproteins. A similar change is observed in the chiral PSP polarization (Supplementary Note 7 and Supplementary Fig. 8A), where the N–H peak vanishes at high humidity. The absence of the N–H peak at high humidity in both SSP and PSP polarizations suggests that the α-helix or β-sheet structures present at the contact interface at 10% RH are altered at high humidity. This could be due to the disruption of intramolecular H-bonds by water harvested by pristine aggregate glue at high humidity^27^. However, these protein rearrangements show reversible behavior because the N–H peak is recovered, as the humidity is cycled back to 10% RH (Supplementary Figs 8A and 9). Future studies will focus on collecting the SFG spectra collected in amide I region that may elucidate which parts of the secondary structure (α-helices or β-sheets) change with increasing humidity.

The washed aggregate glue is expected to be less humidity-responsive than the pristine aggregate glue due to the absence of LMMCs, as reflected in a smaller change in the refractive index of washed aggregate glue relative to pristine aggregate glue (Supplementary Table 2). The washed aggregate glue/sapphire SFG spectrum collected in PPP polarization undergoes only minor changes in the D_2_O region as humidity is increased from 10 to 90% RH (Fig. 4c). This is consistent with our expectation in that the washed aggregate glue shows only ~20% water uptake in contrast to a ~100% water uptake for pristine aggregate glue^33^. The minimal responsiveness of washed aggregate glue to increasing humidity is also apparent in the ATR-IR spectra of washed aggregate glue collected at different humidities (Fig. 5b). In comparison to pristine aggregate glue SFG spectrum in SSP polarization, the SSP spectrum for washed aggregate glue at high humidity (Fig. 4d) shows features similar to the one at low humidity, where a strong N–H peak is observed at ~3260 cm^−1^. These observations indicate that with humidity, changes in the protein secondary structures do not occur in the washed aggregate glue. Similar results have been reported previously with solid-state NMR, where the molecular mobility is lost at high humidity after removal of hygroscopic LMMCs from pristine aggregate glue^20^.

## Discussion

We utilize interface-sensitive SFG in conjunction with ATR-IR to provide direct evidence of glycoproteins at the contact interface, as previously hypothesized in literature^7, 14, 24–27^. Glycosylation occurs in glues produced by many organisms^5, 6^ and is therefore a common strategy in nature to design adhesive proteins. However, the glycoproteins by themselves (as in washed aggregate glue) do not stick well as the removal of LMMCs results in an irreversible change in glycoproteins, thus highlighting the critical role played by LMMCs in forming a good initial contact^20^. Previous studies show that LMMCs keep the glycoproteins soft and mobile by sequestering atmospheric water and directly solvating the glycoproteins^20^. Our results show that LMMCs play a key role not only in facilitating initial contact but also in maintaining adhesion, especially at high humidity. At 90% RH, liquid-like water is absent from the contact interface (Fig. 4a and Supplementary Fig. 3), although the aggregate glue contains 50% liquid-like water in the bulk (Fig. 5a)^33^. If liquid-water were strongly bonded with the substrate at high humidity, it would result in de-bonding of the aggregate glue from the substrate, which is not observed experimentally^49^. In addition, the insensitivity of the sapphire interaction peak (~3550 cm^−1^) to increase in humidity shows that the bonds between the aggregate glue and sapphire surface remain intact as humidity increases. We observe reversible changes in the secondary structures of glycoproteins present at the interface at high humidity for pristine aggregate glue suggesting water–protein interactions. In contrast to pristine aggregate glue, liquid-like water is observed at the polyacrylic acid/sapphire interface at high humidity. Our SFG observations of pristine aggregate glue together with results obtained for model polyacrylic acid polymer suggest the sequestration of interfacial liquid-like water by the hygroscopic LMMCs, thus explaining the cohesive failure of *Larinioides cornutus* aggregate glue at high humidity. Tan et al. reports similar observations with synthetic adhesives, where the osmotic pressure effect of sodium chloride in water improves the adhesion underwater^50^. Thus, hygroscopic LMMCs and glycoproteins team-up in sequestering interfacial water at high humidity, thereby overcoming a fundamental limitation of conventional synthetic adhesives.

In summary, orb-web spiders use a complex assembly of glycoproteins and hygroscopic LMMCs in aggregate glue droplets to adhere prey. Spider aggregate glue is humidity-responsive and resists interfacial failure under high humidity. Here, we use interface-sensitive SFG to show that glycoproteins act as primary binding agents, while the hygroscopic LMMCs preserve the bonds between the aggregate glue and the substrate by actively sequestering liquid-like water away from the interface thereby preventing interfacial failure at high humidity. Interfacial water sequestration by means of hygroscopic additives can be a powerful design strategy to avoid interfacial failure in synthetic adhesives at high humidity.

## Methods

### Spider care and sample collection

*Larinioides cornutus*, common orb-weaving spiders, were collected in Akron, OH, USA and housed in separate cages in a lab environment at ambient humidity. The spiders were fed a weekly diet of crickets and allowed to make webs daily. The capture spiral silk (BOAS) strands were collected from 2–3 fresh webs for SFG and ATR-IR experiments.

### SFG experiment

SFG measurements are carried out using a picosecond Spectra Physics laser system, a detailed description provided in a previous publication^3, 29, 42^. Briefly, it involves the overlap of a fixed 800 nm visible beam (~70 μJ energy, 1 ps pulse width, 1 kHz repetition rate, 1 mm diameter) with a tunable infrared (IR) beam 2000–3800 cm^−1^ (~3.5 μJ energy, 1 ps pulse width, 1 kHz repetition rate, 100–200 μm diameter). Due to the use of a picosecond laser system where the visible beam is not focussed, the problem of sample damage is minimal. An equilateral sapphire prism (15 × 15 × 15 × 10 mm, *c*-axis ±2° parallel to the prism face, Meller Optics Inc.) is used as a substrate on which the capture silk (BOAS) is immobilized. The sapphire prism is cleaned by sequential sonication in toluene, acetone, ethanol, and ultrapure water (18.2 MΩ cm from a Millipore filtration system, pH 6–7) for 1 h each with drying in between using a N_2_ jet. Finally, the prism is plasma sterilized (Harrick Plasma, PDC-32G) and mounted onto a clean stainless steel sample holder to collect a blank scan. After collecting a blank scan, the sapphire prism is removed from the sample holder and multiple BOAS (capture silk) strands are adhered to it in a parallel configuration (Fig. 1a, b). The sapphire prism with multiple BOAS strands is mounted on to a sample holder and exposed to high humidity (90% RH) to allow complete spreading of aggregate glue droplets resulting in the formation of uniform aggregate glue layer (Fig. 1c)^10, 12, 26^. The aggregate glue in its native form has been referred to as pristine aggregate glue in the text. The sapphire prism with pristine aggregate glue is further placed in contact with Millipore water for 1 h to remove the water soluble LMMCs and leave washed aggregate glue containing only glycoproteins. The efficacy of washing is tested by collecting a Raman spectrum of the washed aggregate glue, which confirms the absence of LMMCs in washed aggregate glue (Supplementary Fig. 2 and Supplementary Note 2). The aqueous LMMC-extract is stored in a sealed vial for further experiments. SFG spectra are acquired for pristine and washed aggregate glue at 10 and 90% RH (using both D_2_O and H_2_O vapors) in total internal reflection (TIR) geometry (favored by the high refractive index of sapphire) using an in situ humidity setup, where the incident angle of the IR beam is set to probe the buried aggregate glue/sapphire interface^51^. The incident angles are determined using linear reflectivity measurements (Supplementary Fig. 10, Supplementary Table 2, and Supplementary Note 8). The TIR geometry significantly enhances the SFG signal, however, it could lead to uneven enhancement of spectral features across the entire scanning IR wavelength^52^. An incident angle of 2° is used to probe contact interface of both pristine and washed aggregate glue with sapphire surface at 10% RH. However, due to differential water uptake by pristine and washed aggregate glue at 90% RH, incident angles of 10° and 6° are used for probing pristine aggregate glue/sapphire and washed aggregate glue/sapphire interfaces at 90% RH, respectively. The incident angle for the visible laser beam is ~1.5° lower than that of the IR beam. Additional SFG spectra were collected for aggregate glue/air interface using 42° incident angle, which appear to be very different from the buried aggregate glue/sapphire SFG spectra (Supplementary Fig. 12 and Supplementary Note 10). A 30 min equilibration time is provided before collecting SFG spectra at respective RH with both D_2_O and H_2_O vapors (Supplementary Note 9). This time window is sufficient for diffusion of water through the aggregate glue to the buried interface (Supplementary Fig. 11). The SFG spectra have been collected in three regions (2200–2800 cm^−1^, 2750–3100 cm^−1^, and 3000–3800 cm^−1^) separately and finally stitched together, where each of the individual scans takes about 15–20 min. The lack of changes in the observed SFG spectra over time indicate that enough time is provided to allow complete H/D exchange (Supplementary Figs 8A and 9). The SFG spectra are collected in PPP, SSP, and PSP polarizations, where the three indices correspond to the polarizations of SFG, visible, and IR beams, respectively. S and P polarization relate to the direction of electric field being perpendicular and parallel respectively to the plane of incidence. The reported SFG spectra have not been corrected for changes in Fresnel factors and have been fitted using a Lorentzian equation (Eq. 1).

1 $$I_{\mathrm{SFG}} \propto \left| {\chi _{\mathrm{NR}} + {\sum} {\frac{{A_q}}{{\omega _{\mathrm{IR}} - \omega _q + i\Gamma _q}}} } \right|^2,$$

In Eq. 1, *χ*_NR_ describes the non-resonant contribution. A_*q*_, Γ_*q*_, and *ω*_*q*_ are the amplitude strength, damping constant, and resonant frequency of the *q*th vibrational resonance, respectively.

### ATR-IR experiment

ATR-IR measurements are carried out using Thermo Nicolet 6700 Fourier transform infrared spectrometer equipped with a Mercury-Cadmium-Tellurium detector. Germanium (Ge) crystal (refractive index = 4.0) with an angle of incidence of 45°, allowing 10 bounces in ATR geometry, is used as a substrate on which the capture spiral silk strands (BOAS)  are immobilized. The crystal is thoroughly cleaned by ultra-sonication in toluene, acetone, and ethanol for 30 min each with drying by N_2_ jet during each solvent change. Finally the crystal is plasma sterilized for 5 min (Harrick Plasma, PDC-32G). The Teflon-coated fluid flow cell (Pike Technologies) containing the ATR crystal is connected with the humidity control system. Different background spectra are obtained at 10%, 50%, and 90% RH controlled by D_2_O . D_2_O is used instead of H_2_O to avoid complications in the deconvolution of overlapping peaks, O–H stretch and amide A/B bands in the 3000–3400 cm^−1^ region. The O–D stretch of D_2_O appears in the 2400–2700 cm^−1^ region, which does not have many peaks from other chemical bond entities. Replacing H_2_O with D_2_O is a common spectroscopic technique used to probe the water structure in synthetic and natural systems. After backgrounds collection, the crystal is removed from the flow cell and the multiple BOAS strands are mounted with special care taken to avoid collecting any other type of silk from the web. Once the crystal containing multiple BOAS strands is mounted back in the flow cell, the sample is in situ conditioned at 90% RH for 5 min allowing complete spreading of aggregate glue droplets and exchange of the native H_2_O content by D_2_O. The aggregate glue was then dried to 10% RH to begin spectral collection. Then with each humidity cycle, the sample is equilibrated at 10%, 50%, and 90% RH for 5 min before spectra are collected at the respective RH. The ATR-IR spectra for pristine aggregate glue do not change over time indicating complete H/D exchange (Supplementary Fig. 7). A comparison of ATR-IR spectra for BOAS (aggregate glue + fiber) and only aggregate glue (no fiber)  is shown in Supplementary Fig. 13 and Supplementary Note 11. The measurements involve Happ–Genzel apodization based on 500 interferograms with spectral resolution of 4 cm^−1^. The absorbance spectra obtained are corrected by automated baseline and advanced ATR correction using Thermo Scientific OMNIC 9.4.251 software.

### Data availability

The authors declare that the data supporting the findings of this study are available from the corresponding author on request.

## Electronic supplementary material

Supplementary Information
