# Supplementary material for: Hygroscopic compounds in spider aggregate glue remove interfacial water to maintain adhesion in humid conditions
Source: Nat Commun. 2018 May 22;9:1890. doi: 10.1038/s41467-018-04263-z (PMC5964112; doi:10.1038/s41467-018-04263-z)
Supplement: Supplementary file 1 — Supplementary Information [file 41467_2018_4263_MOESM1_ESM.pdf]

Supplementary Information for:

Hygroscopic Compounds in Spider Aggregate Glue Remove Interfacial Water to Maintain Adhesion in Humid Conditions

Saranshu Singla[1,5], Gaurav Amarpuri[1,3,5], Nishad Dhopatkar[1,4], Todd A. Blackledge[2], Ali Dhinojwala[1,6]

[1]*Department of Polymer Science, The University of Akron, Akron, Ohio 44325, United States*
[2]*Department of Biology, Integrated Bioscience Program, The University of Akron, Akron, Ohio 44325, United States*
[3]*Currently at Eastman Chemical Company, Corporate Analytical Division, Kingsport, TN 37662, United States*
[4]*Currently at Avery Dennison Polymers, Adhesives and Coatings Center of Excellence, Mill Hall, PA 17751, United States*
[5]*Both authors contributed equally to the paper*
[6]*Email:ali4@uakron.edu*

Supplementary Note 1

SFG Spectra for Pristine and Washed Aggregate Glue at 10% RH After Exposing to D_2O Vapors

The component peaks used for fitting the SFG spectra of pristine and washed aggregate glue at buried interface in PPP, SSP and PSP polarizations (Fig. 2 and 3) have been shown using blue dashed lines in Supplementary Fig. 1.

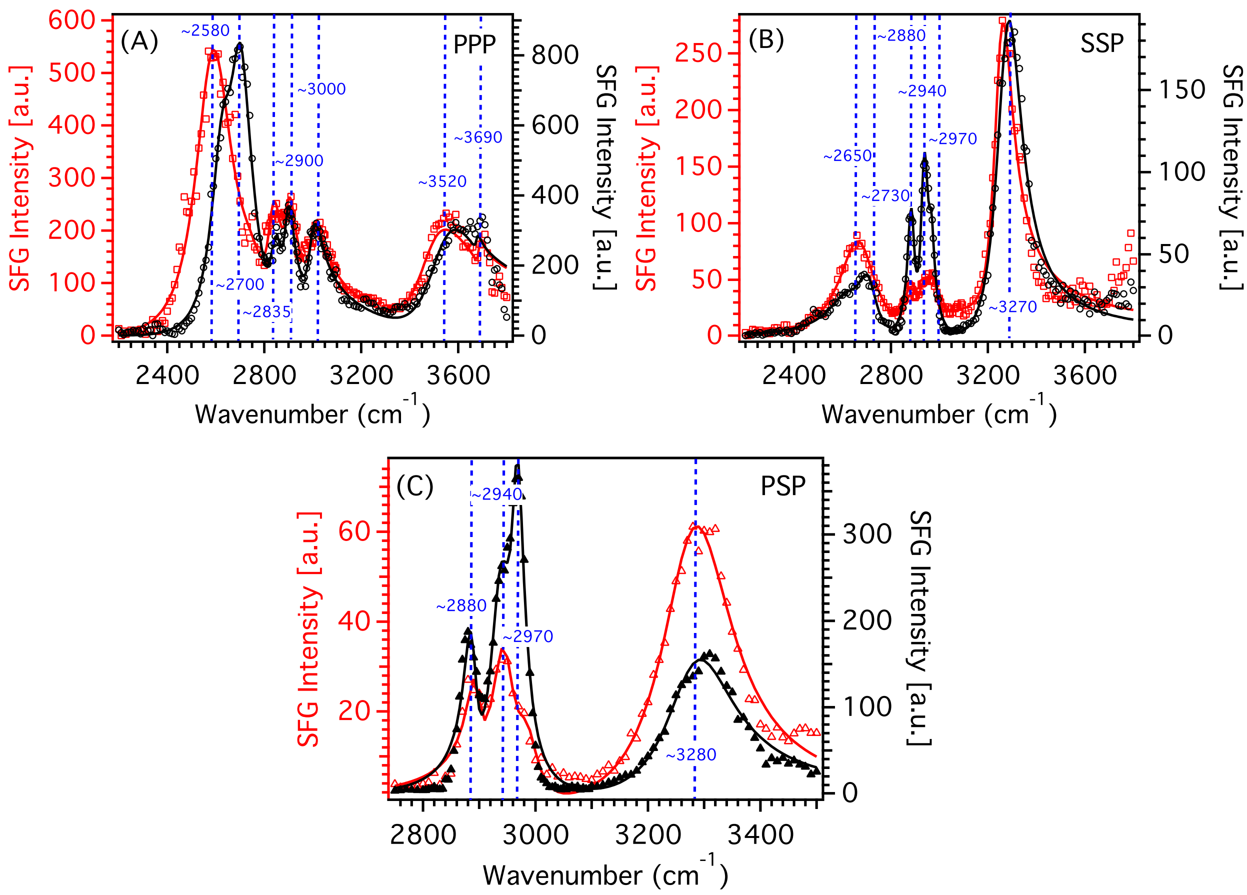

Supplementary Figure 1. SFG spectra collected for pristine (red empty squares) and washed (black empty circles) aggregate glue in PPP (A) and SSP (B) polarizations at 10% RH collected using D_2O vapors. SFG spectra collected for pristine (red empty triangles) and washed (black solid triangles) aggregate glue in PSP polarization at 10% RH collected using D_2O vapors (C). The solid lines are fit to the Lorentzian equation and the component peak positions are marked by blue dashed lines.

Supplementary Note 2

Raman Spectrum of Pristine and Washed Aggregate Glue

The sapphire prism with pristine aggregate glue is placed in contact with water for ~1 h to remove the water-soluble low molecular mass compounds (LMMCs). The washing process is expected to irreversibly remove the LMMCs and leave washed aggregate glue containing only the glycoproteins as previously shown using NMR.[1] The efficacy of the washing process is tested by collecting Raman spectra of aggregate glue before (pristine) and after washing (washed) using a LabRam HR Micro Raman Spectrometer (Horiba) coupled to a Olympus BX41 motorized stage microscope (Supplementary Fig. 2).[2] The washed aggregate glue Raman spectrum (black curve) does not show the LMMC-specific peak (indicated by blue dashed box), which is present in the pristine aggregate glue Raman spectrum (red curve), confirming our claim that only glycoproteins are left behind after washing.

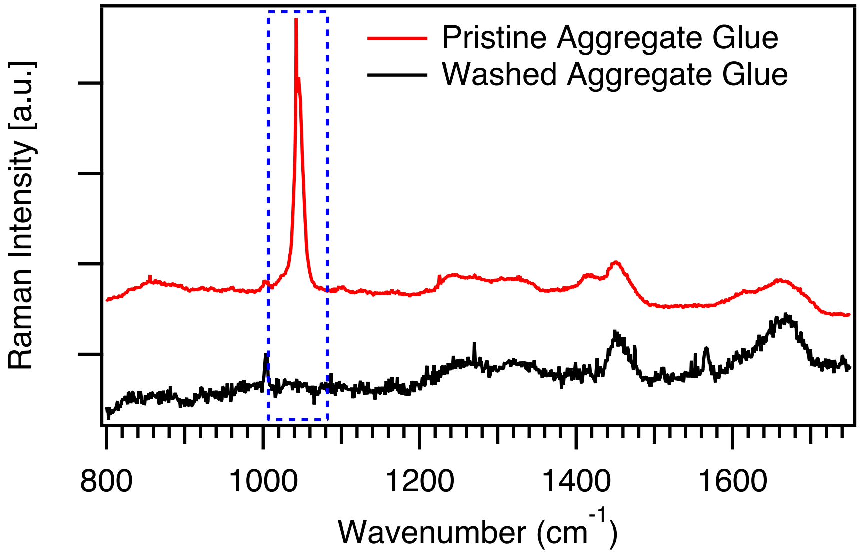

Supplementary Figure 2: Raman spectra of pristine (red curve) and washed (black curve) aggregate glue. The spectra have been vertically offset for clarity. The absence of LMMC-specific peak (indicated by blue dashed box) in the washed aggregate glue spectrum indicates that washed aggregate glue consists of only glycoproteins.

Supplementary Note 3

SFG Spectra Collected After Exposing to H_2O Vapors

(a) Pristine Aggregate Glue

Supplementary Fig. 3 presents the SFG spectra for pristine aggregate glue/sapphire interface at 10%, 50% and 90% RH using H_2O vapors collected in PPP polarization at an incident angle of

2°. The SFG spectra for pristine aggregate glue/sapphire collected at 3 different RH look very similar, except for a drop in overall SFG intensity at 90% RH (Supplementary Fig. 3). The reduction in SFG intensity at 90% RH could be attributed to a change in the refractive index (RI) of pristine aggregate glue at 90% RH (Supplementary Table 2). All the SFG spectra include dominant peaks at ~3560 cm^{-1} and ~3660 cm^{-1}, attributed to the loosely coordinated water (or less hydrogen bonded water),[3-6] and the shifted sapphire O-H due to strong interactions between pristine aggregate glue and sapphire substrate.[7,8] The interaction energy between pristine aggregate glue and sapphire is calculated as a function of RH using the Badger-Bauer equation (equation 1) and tabulated in Supplementary Table 1.[7] A minimal change in the interaction energy with humidity suggests that the adhesive bonds between the pristine aggregate glue and sapphire stay intact at high humidity. In addition, no liquid-like water peak is observed at 90% RH even though pristine aggregate glue shows 100% water uptake inside the bulk.[9]

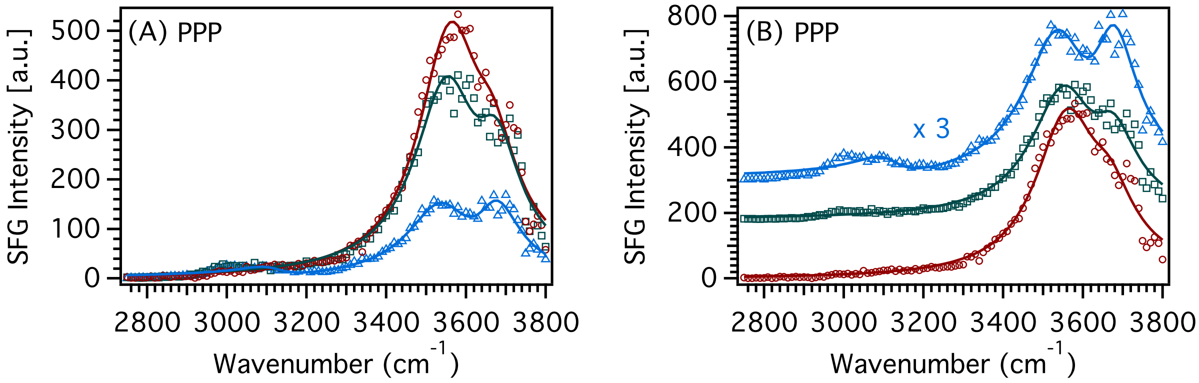

Supplementary Figure 3: SFG spectra collected in PPP polarization for pristine aggregate glue/sapphire interface at 10% (red empty circles), 50% (green empty squares) and 90% (blue empty triangles) RH. Solid lines are fits obtained using a Lorentzian equation. The raw SFG spectra are presented in (A). The SFG spectrum collected at 90% RH has been multiplied by a factor of 3 for comparison purposes and the SFG spectra at different humidities have been vertically offset for clarity in (B).

Badger-Bauer Equation

$$\Delta H = m * \Delta \nu + C \qquad (1)$$

In equation 1, m and C represent the slope and intercept of enthalpy, ΔH (kcal/mol) vs. frequency shift, Δν (cm^{-1}) plot. We used literature reported values of m and C for calculating the interaction energies.[7]

(b) Washed Aggregate Glue

Supplementary Fig. 4A, B show the SFG spectra collected for washed aggregate glue/sapphire interface at 10% and 90% RH using H_2O vapors in PPP and SSP polarizations, respectively. The

PPP spectrum (Supplementary Fig. 4A) for washed aggregate glue/sapphire consists of dominant peaks at ~3520 cm^{-1} and ~3650 cm^{-1} similar to pristine aggregate glue/sapphire (Supplementary Fig. 3) along with minor peaks in the C-H stretching vibration region. The SSP spectrum (Supplementary Fig. 4B) for washed aggregate glue/sapphire at 10% RH shows C-H vibration peaks (2890, 2940 and 2960 cm^{-1}) along with a N-H stretch at ~3260 cm^{-1}.[10-14] The N-H peak stays intact as the humidity is increased to 90% RH.

Supplementary Table 1. Interaction energy between pristine aggregate glue and sapphire substrate calculated as a function of RH using the Badger-Bauer equation.[7]

Relative Humidity (%)	Interaction Energy (kcal/mol)
10% RH	1.47
50% RH	1.41
90% RH	1.38

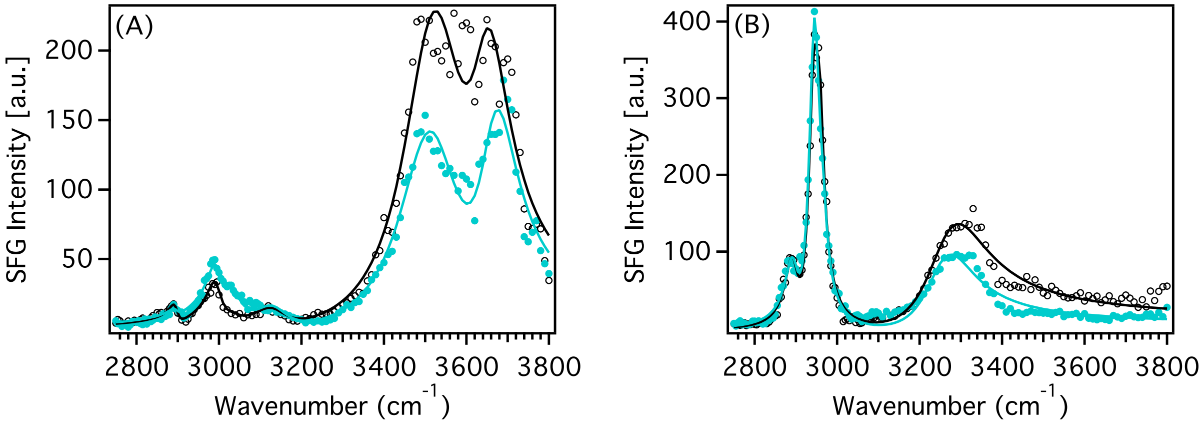

Supplementary Figure 4: SFG spectra collected in PPP (A) and SSP (B) polarizations for washed aggregate glue/sapphire interface after exposing the samples to 10% RH (black empty circles) and 90% RH (blue solid circles) obtained using H_2O vapors. The solid lines are fit to the Lorentzian equation.

Supplementary Note 4

SFG Spectra for LMMC-extract

The LMMC-extract obtained by washing the pristine aggregate glue with water is used for drop-casting a LMMC film on the sapphire prism. The sapphire prism with LMMC-extract film is vacuumed for 15 min before collecting SFG spectra. SFG spectra are collected for the LMMC-extract film at 42° (LMMC-extract/air, red curve) and 10° (LMMC-extract/sapphire, black curve) incident angles, as shown in Supplementary Fig. 5. The SFG spectra collected at two

different incident angles look identical, except for overall changes in SFG intensity, indicating the LMMC-film on the sapphire prism is too thin to differentiate between the two interfaces. The LMMC-extract/sapphire SFG spectra in both PPP and SSP polarizations consist of hydrocarbon peaks in the C-H stretching vibration region along with shifted sapphire-OH peak at ~3600 cm^{-1}.[7,8] Unlike the pristine and washed aggregate glue SFG spectra (Supplementary Fig. 1), the LMMC-extract/sapphire SFG spectrum in SSP polarization does not show a N-H peak, again suggesting that the N-H peak observed for both pristine and washed aggregate glue could be assigned to the glycoproteins.

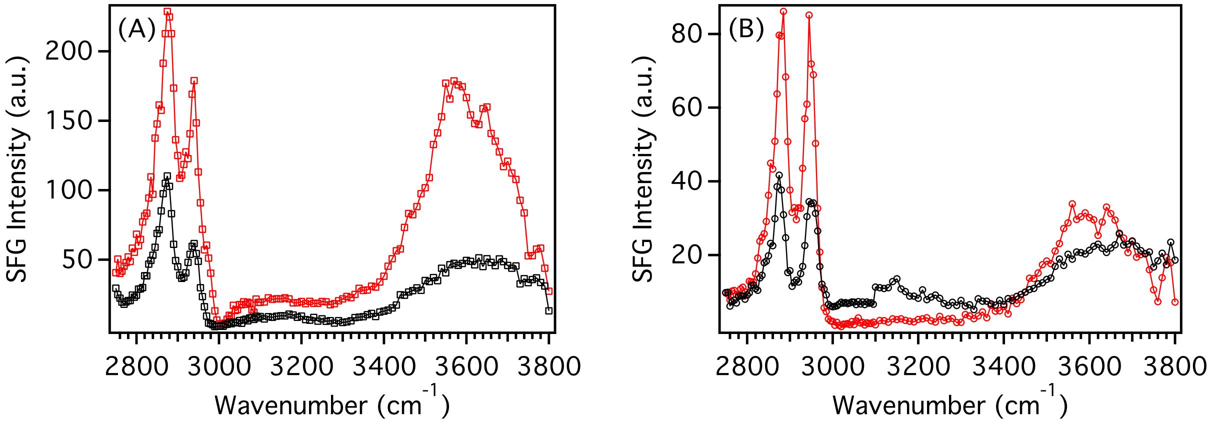

Supplementary Figure 5. SFG spectra collected for LMMC-extract in PPP (A) and SSP (B) polarizations at 42° (red curve) and 10° (black curve) incident angles.

Supplementary Note 5

SFG Spectra for Polyacrylic Acid

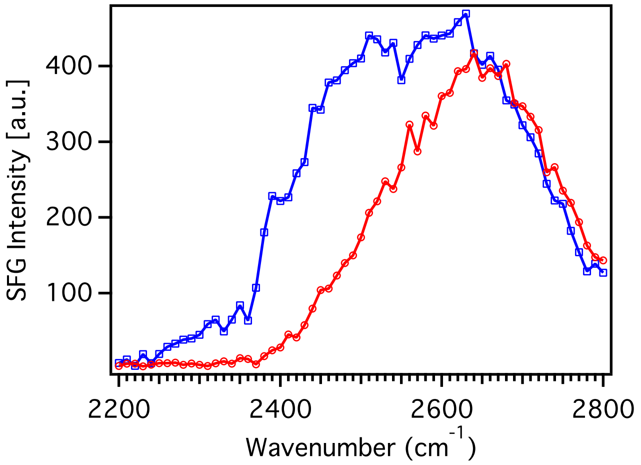

Supplementary Figure 6. SFG spectra collected in PPP polarization for polyacrylic acid/sapphire at 10% RH (red empty circles) and 90% RH (blue empty squares) using D_2O vapors.

A polyacrylic acid (PAA) film spin-coated on sapphire prism is used as a control and subjected to varying humidity conditions using D_2O vapors to determine the water structure at PAA/sapphire interface (Supplementary Fig. 6). The SFG spectra collected at 10% RH predominantly shows the presence of low coordination water, whereas at 90% RH additional liquid-like water peak at ~2400 cm^{-1} is observed. This is in stark contrast to pristine aggregate glue/sapphire, where no liquid-like water peak is observed at 90% RH, although pristine aggregate glue shows a 100% water uptake in the bulk.[9]

Supplementary Note 6

ATR-IR Spectra of Pristine Aggregate Glue

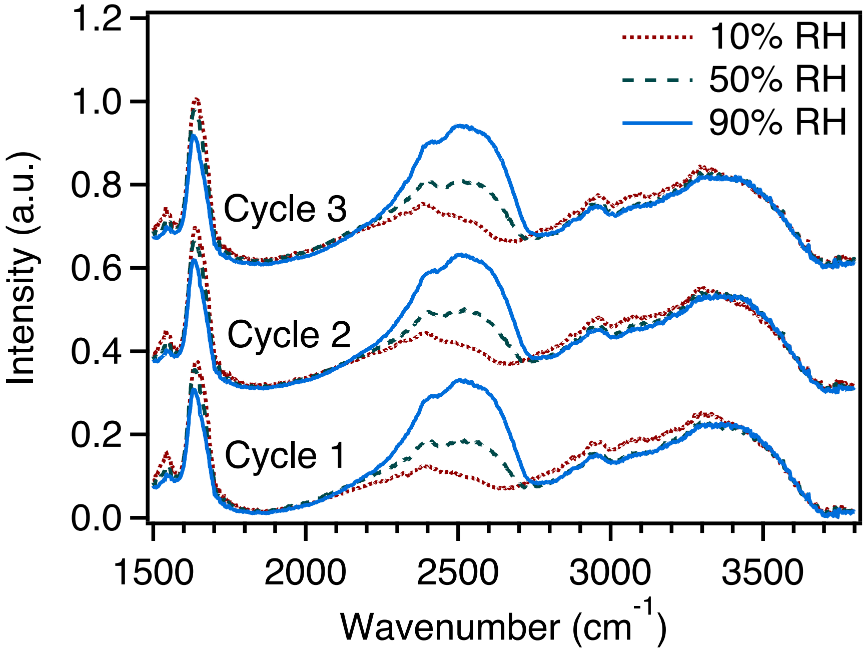

Supplementary Figure 7. ATR-IR spectra of *Larinioides cornutus* pristine aggregate glue at 10% RH (red dotted curve), 50% RH (green dashed curve) and 90% RH (blue solid curve) collected using D_2O vapors during the cyclic measurements (where the humidity is cycled from 10% to 50% to 90% RH and then returned to 10% RH and so on).

Supplementary Fig. 7 shows the ATR-IR spectra of pristine aggregate glue at 10%, 50% and 90% RH collected using D_2O vapors. The ATR-IR spectra at different relative humidities show minor changes in the amide I/II, C-H stretch and N-H/O-H stretch regions, except for changes in the D_2O (2200-2800 cm^{-1}) region which are shown in Fig. 5A in the main text. The minimal change in the amide I/II region suggests that no significant changes occur in the protein conformation in the bulk.

We also cycled the humidity from 10% to 50% to 90% RH and then returned to 10% RH to see if any changes in ATR-IR spectra occur with different exposure time to D_2O vapors. As shown in

Supplementary Fig. 7, the spectra look very similar during the different cycles 1, 2 and 3 suggesting that the presented spectra are collected after allowing complete H/D exchange.

Supplementary Note 7

SFG Spectra for Pristine and Washed Aggregate Glue in PSP Polarization

SFG spectra are collected for pristine and washed aggregate glue at 10% and 90% RH in PSP polarization, which is selective to the presence of chiral structures at the interface (Supplementary Fig. 8). The SFG spectrum for both pristine aggregate glue/sapphire and washed aggregate glue/sapphire interface at 10% RH shows a N-H stretch peak at ~3250 cm^{-1}, assigned to the presence of α-helices or β-sheets in pristine and washed aggregate glue. The N-H peak disappears at 90% RH for pristine aggregate glue/sapphire, whereas the N-H peak stays intact for washed aggregate glue/sapphire interface. The disappearance of N-H peak in PSP is reversible (black curve) as the humidity is reduced again to 10% RH.

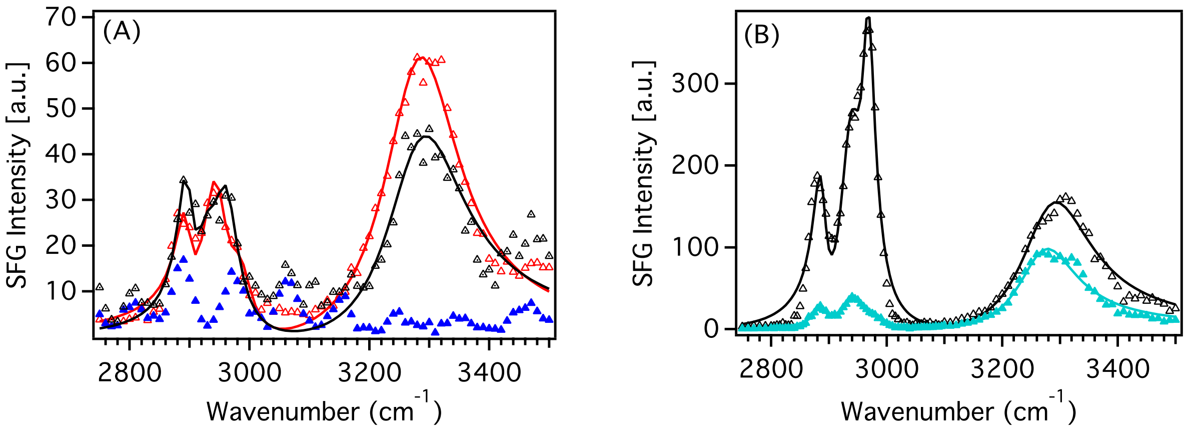

Supplementary Figure 8. SFG spectra collected for pristine aggregate glue/sapphire interface at 10% RH (red empty triangles), 90% RH (blue solid triangles) in PSP polarization and again at 10% RH (black empty triangles) obtained using D_2O vapors (A). Similar SFG spectra collected for washed aggregate glue/sapphire interface at 10% RH (black empty triangles) and 90% RH (blue solid triangles) in PSP polarization (B).

SFG Spectra for Pristine Aggregate Glue in SSP Polarization

We cycled the humidity from 10% RH to 90% RH and returned to 10% RH to see if any changes occur in the SFG spectra with different exposure time to D_2O vapors. Supplementary Fig. 9 shows the SFG spectra collected for pristine aggregate glue/sapphire interface at 10% RH, 90% RH and again at 10% RH in SSP polarization. The SFG spectra at 10% RH collected before and after cycling to 90% RH show similar peaks (the N-H stretch peak at ~3270 cm^{-1} along with C-H stretch peaks) suggesting that the spectra shown in the present study are collected after allowing complete H/D exchange.

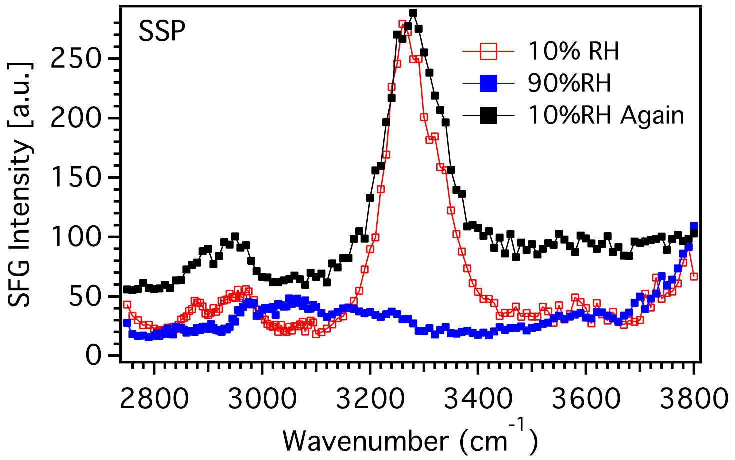

Supplementary Figure 9. SFG spectra for pristine aggregate glue collected at 10% (red empty squares), 90% (blue filled squares) and again at 10% RH (black filled squares) in SSP polarization. The SFG spectrum collected at 10% RH after cycling to 90% RH has been vertically offset for clarity.

Supplementary Note 8

Linear Reflectivity Measurements

Helium-neon reflectivity measurements are used to estimate the RI of pristine and washed aggregate glue, where the reflected intensity is measured as a function of incident angle. Supplementary Fig. 10 shows the normalized intensity plotted as a function of incident angle for pristine aggregate glue at 10% (red empty circles), 50% (black empty triangles) and 90% (blue empty squares) RH. The incident angle at which the intensity changes dramatically corresponds to the critical angle for total internal reflection. The critical angle increases with increasing RH due to a consistent increase in water content inside pristine aggregate glue with increasing RH.[9,15] The RI are calculated for pristine aggregate glue at varying RH using Snell's law with the help of a two-layer model (Supplementary Table 2). The calculated curves (solid lines) reproduce the same trend as experimental data points, except at large values of incident angles. The mismatch between experimental and calculated curves at higher incident angles could be attributed to the spatial heterogeneity of the system. Similar measurements are done with washed aggregate glue (devoid of LMMCs); the values are summarized in Supplementary Table 2.

Supplementary Table 2. Refractive indices (RI) of pristine and washed aggregate glue as a function of humidity calculated using the linear reflectivity measurements.

Sample	10% RH	50% RH	90% RH
Pristine aggregate glue	1.51	1.47	1.39
Washed aggregate glue	1.51	-	1.45

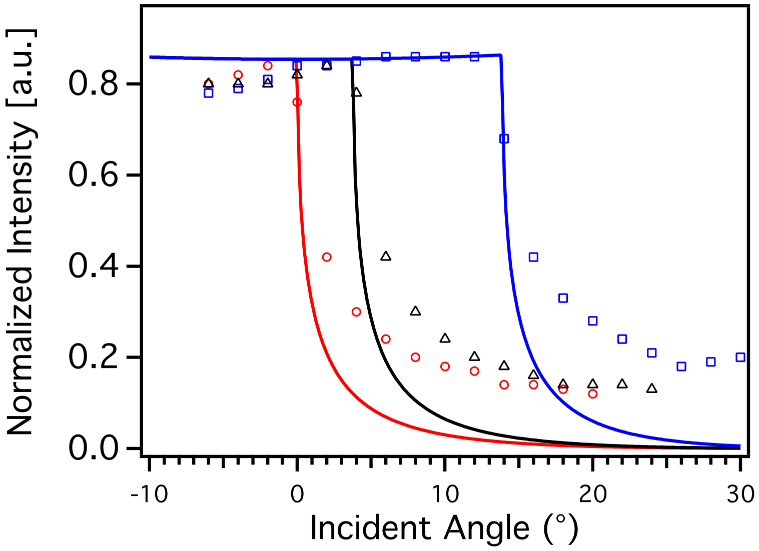

Supplementary Figure 10. Normalized intensity plotted as a function of incident angle for pristine aggregate glue at 10% (red empty circles), 50% (black empty triangles) and 90% (blue empty squares) RH. The solid lines represent the fit obtained using a two-layer model.

Supplementary Note 9

Equilibration Time

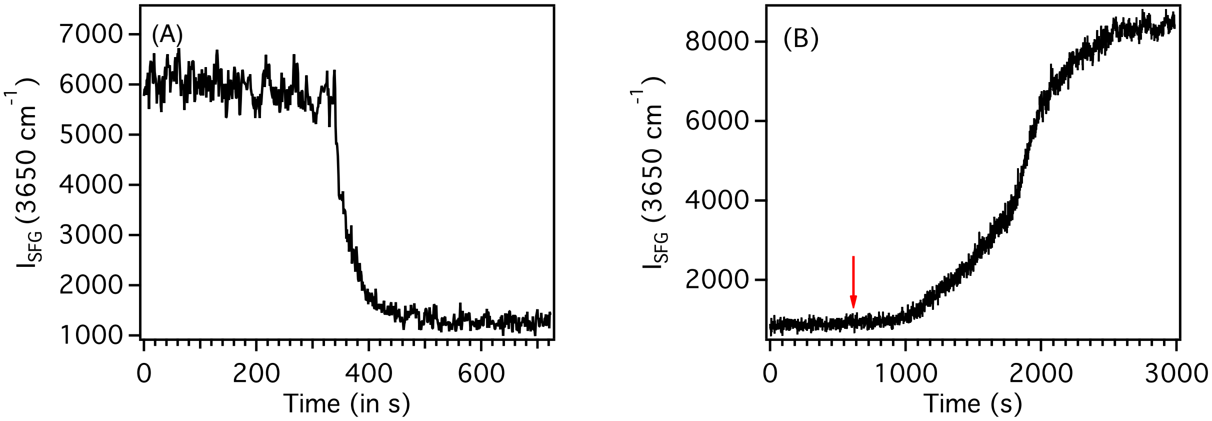

Supplementary Figure 11. SFG intensity at 3650 cm^{-1} in PPP polarization plotted as a function of time as the humidity is increased from 10% to 90% RH (A) followed by decreasing the humidity from 90% back to 10% RH (B). The red arrow indicates the time instant at which the humidity is switched from high to low RH.

The SFG intensity at 3650 cm^{-1} (PPP, Supplementary Fig. 3) is monitored as a function of humidity to measure the time required to humidify (or dehumidify) the pristine aggregate glue on

the sapphire prism. The SFG intensity at 3650 cm^{-1} attenuates quickly as the humidity is increased from 10% to 90% RH (Supplementary Fig. 11A), indicating a quick water uptake by pristine aggregate glue due to the presence of hygroscopic LMMCs. However, as the humidity is subsequently reduced back to 10% RH, the SFG intensity at 3650 cm^{-1} gradually recovers over a period of 30-35 min (Supplementary Fig. 11B). Thus, we use an equilibration time of 30 min before collecting SFG spectra at a particular RH.

Supplementary Note 10

Comparison of Buried vs. Air Interface SFG Spectra

In addition to the SFG spectra for aggregate glue/sapphire interface, we also collected SFG spectra for aggregate glue/air interface at different relative humidities in PPP and SSP polarizations using 42° incident angle (w.r.t. sapphire surface normal). Supplementary Fig. 12 shows a comparison of buried and air interface SFG spectra for pristine aggregate glue at 10% RH obtained using D_2O vapors in SSP polarization. Clearly, the N-H peak observed at the buried aggregate glue/sapphire interface is absent at the aggregate glue/air interface, suggesting the interfacial structure at the buried interface is different from the air interface.

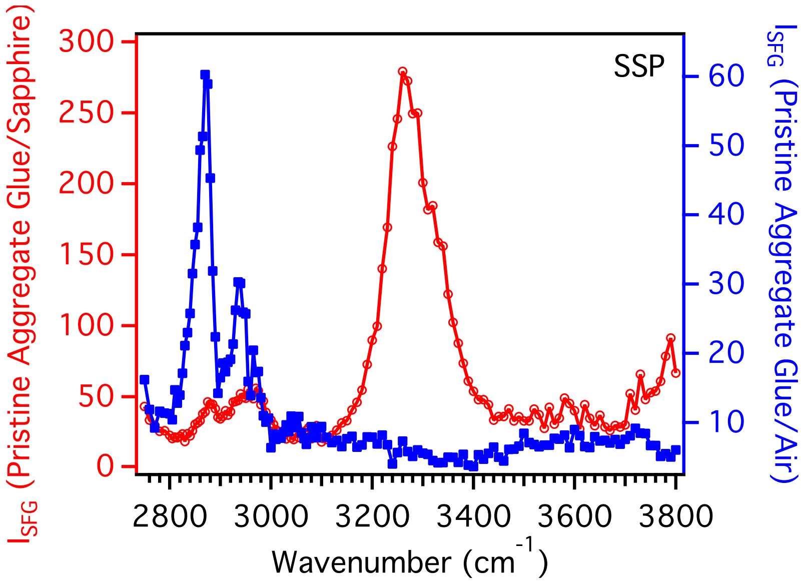

Supplementary Figure 12. Comparison of SFG spectra for pristine aggregate glue collected at 10% RH (obtained using D_2O vapors) in SSP polarization for buried (red empty circles) and air (blue solid squares) interface.

Supplementary Note 11

ATR-IR Spectrum of Spider Aggregate Glue With and Without Underlying Thread

We collected ATR-IR spectra of *Larinioides cornutus* spider aggregate glue with and without the underlying flagelliform thread. To do this, pristine beads-on-a-string (BOAS) were brought in contact with the ATR-IR crystal at 90% RH. When these pristine BOAS were retracted from the surface of ATR-IR crystal at 90% RH, the aggregate glue droplets underwent cohesive failure leaving aggregate glue layer on the crystal (and not the underlying flagelliform thread).[15] ATR-IR spectra were collected for both the pristine BOAS (aggregate glue + flagelliform thread) and aggregate glue shown in Supplementary Fig. 13. The two spectra show similar peaks suggesting the aggregate glue primarily contributes to the observed ATR-IR spectra from BOAS. However, differences in relative peak ratios between the spectra collected with (pristine BOAS) and without the underlying flagelliform thread indicate there may be some contribution from the underlying flagelliform thread in the pristine BOAS ATR-IR spectrum.

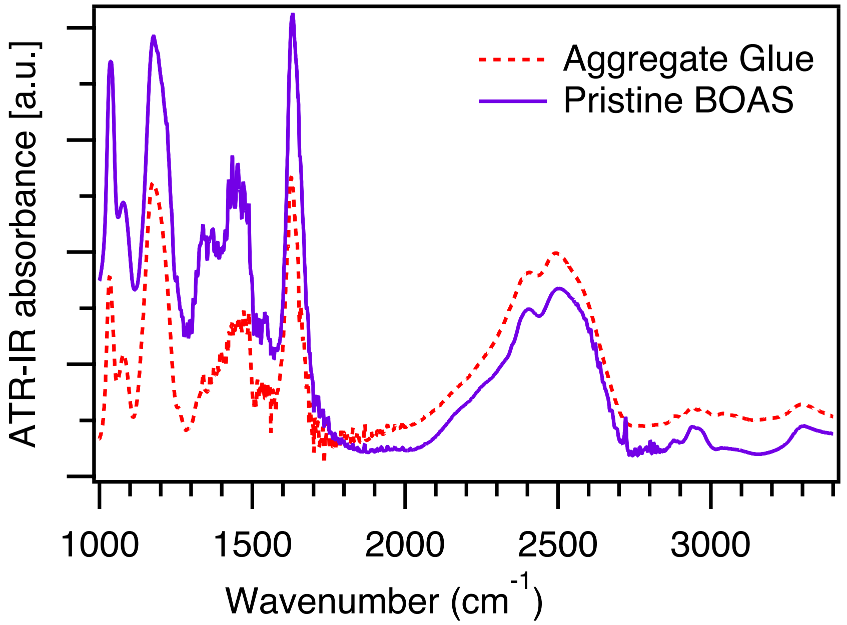

Supplementary Figure 13. ATR-IR spectra of *Larinioides cornutus* aggregate glue collected at 50% RH with (pristine BOAS, purple solid curve) and without (red dashed curve) the underlying flagelliform thread.

Supplementary Note 12

SFG Fitting Parameters

The SFG fitting parameters are provided in Supplementary Tables 3 and 4.

Supplementary Table 3. SFG fitting parameters obtained by fitting the data in Fig. 4A, 4B for the pristine aggregate glue/sapphire interface collected in PPP and SSP polarizations, respectively.

10% RH (PPP)	ω_q/cm^{-1}	2584	2737	2832	2897	2998	3482	3687
	Γ_q/ cm^{-1}	95	74	35	25	34	126	10
	A_q	2123	182	248	132	182	1096	20
90% RH (PPP)	ω_q/cm^{-1}	2594	2733	2834	2880	2960	3488	3689
	Γ_q/ cm^{-1}	95	74	35	25	60	109	10
	A_q	854	454	130	54	339	677	19
10% RH (SSP)	ω_q/cm^{-1}	2650	2729	2873	2928	2967	3250	
	Γ_q/ cm^{-1}	94	75	20	20	20	53	
	A_q	774	105	89	79	69	797	

Supplementary Table 4. SFG fitting parameters obtained by fitting the data in Fig. 4C, 4D for the washed aggregate glue/sapphire interface collected in PPP and SSP polarizations, respectively.

10% RH (PPP)	ω_q/cm^{-1}	2629	2697	2841	2894	2995	3523	3677
	Γ_q/ cm^{-1}	58	54	32	23	34	128	10
	A_q	954	1054	226	177	277	1506	13
90% RH (PPP)	ω_q/cm^{-1}	2632	2716	2832	2891	2987	3515	3686
	Γ_q/ cm^{-1}	70	62	28	22	56	126	10
	A_q	404	392	127	74	319	537	16
10% RH (SSP)	ω_q/cm^{-1}	2623	2709	2886	2938	2965	3277	
	Γ_q/ cm^{-1}	73	53	18	20	14	64	
	A_q	172	246	113	168	44	854	
90% RH (SSP)	ω_q/cm^{-1}	2653	2722	2896	2946	2979	3268	
	Γ_q/ cm^{-1}	76	49	20	20	20	67	
	A_q	154	123	70	122	68	972	

Supplementary References

1. Sahni, V.; Miyoshi, T.; Chen, K.; Jain, D.; Blaimires, S. J.; Blackledge, T. A.; Dhinojwala, A. Direct Solvation of Glycoproteins by Salts in Spider Silk Glues Enhances Adhesion and Helps To Explain the Evolution of Modern Spider Orb Webs. *Biomacromolecules* **2014**, 15, 1225-1232.

2. Amarpuri, G.; Chaurasia, V.; Jain, D.; Blackledge, T. A.; Dhinojwala, A. Ubiquitous Distribution of Salts and Proteins in Spider Glue Enhances Spider Silk Adhesion. *Sci. Rep.* **2015**, 5, 9030.

3. Kusanagi, H.; Yukawa, S. Fourier Transform Infrared Spectroscopic Studies of Water Molecules Sorbed in Solid Polymers. *Polymer* **1994**, 35, 5637-5640.

4. Kitano, H.; Ichikawa, K.; Fukuda, M.; Mochizuki, A.; Tanaka, M. The Structure of Water Sorbed to Polymethoxyethylacrylate Film as Examined by FT-IR. *J. Colloid Interface Sci.* **2001**, 242, 133-140.

5. Morita, S.; Tanaka, M.; Yukihiro, O. Time-Resolved *In Situ* ATR-IR Observations of the Process of Sorption of Water into a Poly(2-methoxyethyl acrylate) Film. *Langmuir* **2007**, 23, 3750-3761.

6. Zhou, J.; Anim-Danso, E.; Zhang, Y.; Zhou, Y.; Dhinojwala, A. Interfacial Water at Polyurethane-Sapphire Interface. *Langmuir* **2015**, 31, 12401-12407.

7. Kurian, A.; Prasad, S.; Dhinojwala, A. Direct Measurement of Acid-Base Interaction Energy at Solid Interfaces. *Langmuir* **2010**, 26, 17804-17807.

8. Zhu, H.; Dhopatkar, N.; Dhinojwala, A. Effect of Acid-Base Interactions on Conformation of Adsorbed Polymer Chains. *ACS Macro Lett.* **2016**, 5, 45-49.

9. Amarpuri, G.; Zhang, C.; Blackledge, T. A.; Dhinojwala, A. Adhesion Modulation Using Glue Droplet Spreading in Spider Capture Silk. *J. R. Soc. Interface* **2017**, 14, 20170228.

10. Weidner, T.; Breen, N. F.; Drobny, G. P.; Castner, D. G. Amide or Amine: Determining the Origin of the 3300 cm^{-1} NH Mode in Protein SFG Spectra Using 15N Isotope Labels. *J. Phys. Chem. B* **2009**, 113, 15423-15426.

11. Fu, L.; Xiao, D.; Wang, Z.; Batista, V. S.; Yan, E. C. Y. Chiral Sum Frequency Generation for *In Situ* Probing Proton Exchange in Antiparallel β-Sheets at Interfaces. *J. Am. Chem. Soc.* **2013**, 135, 3592-3598.

12. Yan, E. C. Y.; Fu, L.; Wang, Z.; Liu, W. Biological Macromolecules at Interfaces Probed by Chiral Vibrational Sum Frequency Generation Spectroscopy. *Chem. Rev.* **2014**, 114, 8471-8498.

13. Okuno, M.; Ishibashi, T. Heterodyne-Detected Achiral and Chiral Vibrational Sum Frequency Generation of Proteins at Air/Water Interface. *J. Phys. Chem. C* **2015**, 119, 9947-9954.

14. Meister, K.; Lotze, S.; Olijve, L. L. C.; DeVries, A. A.; Duman, J. G.; Voets, I. K.; Bakker, H. J. Investigation of the Ice-Binding Site of an Insect Antifreeze Protein Using Sum-Frequency Generation Spectroscopy. *J. Phys. Chem. Lett.* **2015**, 6, 1162-1167.

15. Amarpuri, G.; Zhang, C.; Diaz, C.; Opell, B. D.; Blackledge, T. A.; Dhinojwala, A. Spiders Tune Glue Viscosity to Maximize Adhesion. *ACS Nano* **2015**, 9, 11472-11478.
